# Adolescent utilization of school based mental health services in the United States

**DOI:** 10.1186/s13033-025-00684-8

**Published:** 2025-08-20

**Authors:** Laura Grunin, José A. Pagán, Gary Yu, Allison Squires, Sally S. Cohen

**Affiliations:** 1 NYU Rory Meyers College of Nursing, 433 1st Avenue, New York, NY 10010 United States; 2 NYU School of Global Public Health, 708 Broadway, New York, NY 10003 United States; 3 Columbia University, 722 West 168th St, New York, NY 10032 United States

**Keywords:** Adolescent, Structural equation model, School-based mental health services

## Abstract

**Background:**

Over 14 million adolescents have a diagnosable mental, behavioral, or emotional disorder yet only 20% receive adequate mental health services. There is a critical need to identify accessible and effective pathways to treatment. School based mental health services (SBMHS) are an optimal setting for timely identification, effective management, and convenient delivery of evidence-based mental health care.

**Methods:**

Using data from the 2019 National Survey on Drug Use and Health, we employed structural equation modeling to examine the relationships between utilization of SBMHS and school and academic engagement, religiosity, self-reported depressive symptoms, and parental monitoring and support.

**Results:**

Higher levels of parental monitoring and support (ß = -0.044, *p* < 0.05) and religiosity (ß = -0.027, *p* < 0.05) along with lower levels of school and academic engagement (ß = 0.069, *p* < 0.001) were associated with decreased adolescent utilization of SBMHS. Adolescents reporting a higher number of depressive symptoms on a scale of 1 to 9 (ß = 0.221, *p* < 0.001) were more likely to utilize SBMHS compared to their counterparts. Self-reported depressive symptoms mediated the relationship between all latent variables (parental monitoring and support [ß = -0.222, *p* < 0.001]; religiosity [ß = -0.051, *p* < 0.001]; school and academic engagement [ß = -0.067, *p* < 0.001]) and adolescent utilization of SBMHS.

**Conclusion:**

Findings from this study offer psychologists, teachers, counselors, school nurses, and administrators specific determinants of service use that can be used to develop strategies for adolescent mental health assessment, increase SBMHS utilization among those in need, and support overall emotional well-being.

**Supplementary Information:**

The online version contains supplementary material available at 10.1186/s13033-025-00684-8.

## Background

Concerns about adolescent mental health were on the rise before the COVID-19 pandemic. In the post-COVID-19 era, many clinicians and public health experts have called attention to a mental health crisis among adolescents in the United States (U.S.) [1, 2]. Approximately 14 million youth and adolescents have a diagnosable mental, behavioral, or emotional disorder [3, 4]. In 2021, over 20% of adolescents, aged 12 to 17, reported at least one major depressive episode over the past year [5], more than 40% felt persistently sad or hopeless (a precursor for major depressive episodes), and 22% seriously considered attempting suicide [6]. Moreover, the prevalence of middle and high school age adolescents reporting depression was almost five times more than elementary school children, aged 6 to 11 [7]. Because half of all lifetime mental health disorders start before an adolescent reaches 14 years of age [8, 9], early screening and intervention are important to mitigate the potentially lifelong consequences of inadequate or no treatment (e.g., poor academic achievement, juvenile delinquency, substance abuse, and impaired physical health) [10–12].

Schools are an optimal setting for timely identification, effective management, and convenient delivery of mental health services. For some adolescents, school based mental health services (SBMHS) may be the only option for receiving treatment due to lack of resources, family concerns, or stigma, and mandatory school attendance means students in need are already at the location where services are provided [13, 14]. SBMHS are programs or interventions delivered by a trained school employee or community consultant supporting the mental health and well-being of the student body and staff alike, in a school or school-related building [15–17]. Evidence shows that SBMHS are cost-effective, increase academic achievement, improve prosocial behaviors, and are often perceived to be more acceptable to family than community-based mental health services [13, 16, 18–20]. Comprehensive SBMHS are integrated into an educational setting and provided by mental health professionals such as social workers, nurse practitioners, and psychologists who collaborate regularly with students’ families, teachers, and community health care personnel [16, 21–23].

### Literature review

Previous studies cited in a scoping review have demonstrated independent relationships between the following six factors and adolescent utilization of SBMHS: (a) socio-demographics, (b) religiosity, (c) self-reported depressive symptoms, (d) parental monitoring and support, (e) stigma, and (f) school and academic engagement [24]. Research showed that young, female adolescents living in a home with both parents were more likely to utilize SBMHS than their older, male counterparts living with a single parent [25, 26]. Evidence regarding the relationships between other sociodemographic characteristics (e.g. race, ethnicity, geographic location, health insurance status) and adolescent use of SBMHS varied in that some studies found significant associations between variables and utilization, while others reported none [27–29]. Evidence is also mixed on each of the individual associations between adolescent utilization of SBMHS and religiosity, parental monitoring and support, stigma, and school and academic engagement [24]. Researchers have documented the positive effects of religiosity on adolescent mental health; however, evidence is sparse regarding its direct influence on adolescent use of SBMHS [30–32]. Increased levels of both parental monitoring and support and school and academic engagement have been shown to serve as protective factors against certain mental health conditions such as depression, anxiety, and suicidal behaviors [33, 34]. Nevertheless, one study analyzing racial/ethnic disparities found no statistically significant relationship between adolescent utilization of mental health services in schools and parental monitoring and support [35]. Lastly, studies have consistently demonstrated that the utilization of SBMHS were positively associated with lower levels of stigma [36, 37] and a greater number of self-reported depressive symptoms [25, 38, 39].

### Theoretical framework

The Andersen Model of Health Care Utilization offers a comprehensive framework for understanding and studying the complex interplay of factors that influence an individual’s use of health care services [40, 41]. Andersen’s population characteristics include predisposing factors, enabling factors, and need factors. These features aim to help predict, explain, and understand utilization of health care services among different populations. When applied to adolescent utilization of SBMHS, Andersen’s process model serves as a critical lens through which to examine factors that might be influential. Predisposing factors such as demographics (i.e., age or gender) and social structures (i.e., family configuration or ethnic group) fundamentally shape the health beliefs and attitudes of an adolescent seeking mental health services. Enabling or impeding factors such as religiosity, parental monitoring and support, or school and academic engagement, function as facilitators or barriers specific to SBMHS use. The same factor may be both a facilitator and a barrier, underscoring the complexities involved in adolescent utilization of these services. Lastly, need factors pertain to variables that show a need for health services such as mental health symptoms (i.e., self-reported depressive symptoms or anxiety) or a chronic condition (i.e., asthma or diabetes). The Andersen Model provides structure for quantitatively analyzing the confluence of individual, familial, and contextual factors that influence an adolescent’s use of mental health services in the educational setting.

### Current study

This purpose of this study was to analyze the relationships between school and academic engagement, religiosity, self-reported depressive symptoms, and parental monitoring and support with utilization of school based mental health services among adolescents using national survey data. We hypothesize the following:

1. Higher levels of parental monitoring and support and religiosity along with lower levels of school and academic engagement are likely to decrease adolescent utilization of SBMHS.
2. Adolescents who reported a higher number of depressive symptoms are more likely to utilize SBMHS compared to their counterparts who report a lower number.
3. Depression mediates the effects of parental monitoring and support, religiosity, and school and academic engagement on SBMHS utilization.

## Methods

### Survey design and participants

The U.S. Substance Abuse and Mental Health Services Administration (SAMHSA) collected nationally and state representative data for the 2019 National Survey on Drug Use and Health (NSDUH) to examine mental health and substance [42]. SAMHSA employs a multistage probability design annually to achieve target sample sizes within each stage to accurately represent the larger population. After the stratification and selection of four sampling stages (census tracts, block groups, segments, and dwelling units), individuals are chosen based on eligibility criteria by a trained field interviewer (FI). Parental informed consent and all youth data were collected on an electronic device by the FI using computer-assisted personal interviewing (CAPI) with at least one parent present for the 2019 survey [43]. The New York University Institutional Review Board did not require review of this study because data are publicly available, de-identified as per NSDUH protocol, and therefore not human subjects research [44].

Noninstitutionalized, civilian individuals older than 12 years of age living in the U.S. were surveyed for the 2019 NSDUH (*N* = 67,625). Participants included civilians on military bases, undomiciled people living in long-term hotels or shelters, residents of college dormitories, group homes, or halfway houses, and those in residential homes across all 50 states and the District of Columbia. As per NSDUH protocol, 25% of the 2019 sample was required to be youth 12 to 17 years old (*n* = 13,397), the ages included in this study [45]. Although more recent data is available, the 2019 survey contains data from the last complete year before youth, their schools, and families were affected by the COVID-19 pandemic and will serve as a comparator for future work.

### Measures

#### Covariates

Covariates included age category (12–13, 14–15, or 16–17), sex (male/female), combined race/ethnicity (White, Black, Hispanic, Asian, and Other [includes Native American, Hawaiian or Pacific Islander, and multiracial]), number of parents/caregivers living in household (0, 1 or 2), health insurance status (none, private, or public), family income category (<$20k, $20k-$49,999, $50k-$74,999, or >$75k), and metropolitan area size (large [total population > 1 million], small [total population < 1 million], or nonmetro).

#### Dependent variable

School based mental health service utilization was measured by the adolescent’s answer to the question: “During the past 12 months…did you receive any treatment or counseling from a school social worker, a school psychologist, or a school counselor for emotional or behavioral problems that were not caused by alcohol or drugs?” Responses were dichotomized and coded as “yes” or “no.”

#### Independent variables

The parental monitoring and support scale (coded 1 to 8) was measured and calculated with a focus on the past 12 months, by the following eight items: (a) Parents check that your homework is done; (b) Parents help with your homework; (c) Parents make you do chores around the house; (d) Parents limit the amount of television you are allowed to watch; (e) Parents limit the time you are allowed out during the school week; (f) Parents let you know when you’d done a good job; (g) Parents tell you they are proud of something you’ve done; and (h) Have you had 5 or more fights with at least one of your parents? The first seven prompts were coded as “no” if responses were seldom or never; if the response was always or sometimes, the answer was coded as “yes.” The last prompt was dichotomized and coded as yes/no.

Five questions/prompts were given to the adolescent exploring the importance of religion in their life for the past 12 months. The religiosity scale (coded 1 to 5) was calculated from responses to the following: (a) Have you participated in faith-based activities? (b) Have you attended religious services? (c) Religious beliefs are important to me; (d) Religious beliefs influence my decisions; and (e) It is important my friends share religious beliefs. If the adolescent did not participate in at least one faith-based activity nor attended at least one religious service, their response was coded, as “no,” otherwise their response was coded as “yes.” The last three prompts were coded as “no” if the response was disagree or strongly disagree; if the answer was agree or strongly agree, the response was coded as “yes.”

The following questions about the past 12 months formed the school and academic engagement scale (coded 1 to 5): a) Have you liked going to school overall? (b) How often have you felt schoolwork is meaningful? (c) Have you felt things learned will be important? (d) Have you felt courses at school are interesting? and (e) How often did your teacher say you are doing a good job? If the adolescent answered that they didn’t like or hated school overall, their response was coded as “no,” otherwise it was “yes.” If the second question was answered seldom or never, the response was coded as “no,” otherwise it was “yes”. If the adolescent answered the third and fourth questions with very or somewhat unimportant, their response was coded as “no,” otherwise it was coded “yes.” Lastly, if the answer was seldom or never for how often the teacher says they are doing a good job, the response was coded as “no.” If their response was always or sometimes, the response was coded as “yes.”

#### Mediating variable

Nine questions based on criteria from the Diagnostic and Statistical Manual of Mental Disorders (5th ed.) [46] and adaptations from the National Comorbidity Survey-Adolescent assessed depressive symptoms [45]. Specifically, adolescents were asked if they had felt any of the following nine symptoms for a two-week period or longer within the past year: (a) sad/empty/depressed most of day or discouraged; (b) lost interest/pleasure in most things; (c) changes in appetite or weight; (d) sleep problems; (e) others noticed they are restless or lethargic; (f) felt tired/low energy nearly every day; (g) felt worthless nearly every day; (h) inability to concentrate or make decisions; and (i) any thoughts or plans of suicide. Each of the nine responses were dichotomized and coded as “yes” or “no.” A depression scale was created for analysis by summing the total “yes” responses (coded 1 to 9). Adolescents with at least five of the nine symptoms on the depression scale and the inclusion of either experienced a depressed mood or a loss of interest or pleasure in most things met criteria for a Major Depressive Episode (MDE) [46].

### Data analysis

Descriptive and correlation statistics were calculated in Stata/SE 15.1 to summarize sociodemographic characteristics and measure associations between latent and outcome variables [47]. Confirmatory factor analysis and structural equation modeling were estimated in MPlus8 to check suitability of the measurement models and present standardized regression coefficients quantifying the relationships between variables [48]. The mediation effect of depression was confirmed using a series of regression models. Statistical regression modeling was used to control for confounding effects of demographic covariates. Person-level sampling weights were applied to yield accurate population estimates represented by the sample. A biostatistician confirmed accuracy of all analyses. Goodness of fit indices for the final structural equation model (SEM) included a Comparative Fit Index (CFI) for which values over 0.90 indicated a strong model fit, a Tucker Lewis Index (TLI) for which values over 0.90 indicated a good fit, a Root Mean Square Error of Approximation (RMSEA) for which values less than 0.05 indicated a tight match, and a Standardized Root Mean Square Residual (SRMR) for which values of 0.09 or less demonstrated a good fit [49]. In line with MPlus8 recommendations, Chi-square difference testing was not used as a goodness of fit criteria because the maximum likelihood with robust standard errors (MLR) estimator was employed [50]. Path coefficients were significant at a level of 0.05 or less.

## Results

### Descriptive statistics

Demographic characteristics of the sample are presented in Table 1. Most sample participants were ages 14–15 years of age, male, White, had one parent residing in the household, had private health care insurance, reported an annual family income over $75k, and lived in a large metropolis. Unweighted and weighted percentages are displayed to demonstrate survey adjustments made for accurate representation of the population. Content from the correlation analysis and the associated table can be found in supplemental material.

**Table 1:** Sociodemographic characteristics (*n* = 13,397 and *N* = 24,905,038)

	Sample Frequency (*n*)	Unweighted Percentage (%)	Weighted Percentage (%)
Age (years)			
12–13	4333	32.4	32.7
14–15	4545	33.9	34.1
16–17	4519	33.7	33.2
Sex			
Male	6856	51.2	50.9
Female	6541	48.8	49.1
Race/Ethnicity			
White	6863	51.2	51.5
Black	1781	13.3	13.5
Hispanic	3186	23.8	24.9
Asian	550	4.1	5.3
Other	1017	7.6	4.8
Number of Parents in Household			
0	567	4.2	4.0
1	9009	67.3	69.3
2	3821	25.5	26.7
Insurance Category			
None	859	6.4	7.0
Private	7348	54.9	56.0
Public	5190	38.7	37.0
Income ($)			
< 20k	1997	14.9	14.0
20k – 49,999	3687	27.5	26.3
50k – 74,999	2022	15.1	14.5
75k+	5691	42.5	45.2
Geography			
Large Metro	5922	44.2	54.5
Small Metro	4793	35.8	31.0
Nonmetro	2682	20.0	14.5

### Measurement model

Confirmatory factor analysis was conducted to validate the measurement model and show individual indicators operationalize each latent variable well. A factor loading of 0.30 indicates a weak correlation whereas results over 0.60 are considered strong [51]. Table 2 shows that all factor loadings on each of the four latent variables of religiosity, parental monitoring and support, school and academic engagement, and depression in the measurement model were statistically significant (*p* < 0.001) and well represented by their observed indicators. All goodness of fit indices indicate a strong measurement model including a CFI equal to 0.901, a TLI equal to 0.895, a RMSEA equal to 0.034, and a SRMR equal to 0.061.

**Table 2:** Measurement model results

Latent Variables	Observed Variables	β
Religiosity (Cronbach’s α = 0.73)		
	Participated in faith-based activity	0.436***
	Attended religious services	0.441***
	Religious beliefs are important	0.785***
	Religious beliefs influence decisions	0.814***
	It is important friends share religious beliefs	0.462***
Parental Monitoring and Support (Cronbach’s α = 0.65)		
	Parents check homework is done	0.524***
	Parents help with homework	0.563***
	Parents make youth do chores around house	0.237***
	Parents limit amount of TV	0.285***
	Parents limit time out during school week	0.259***
	Parents tell youth had done good job	0.724***
	Parents tell youth proud of things done	0.688***
	Had more than 5 fights with at least one of your parents	-0.226***
School and Academic Engagement (Cronbach’s α = 0.79)		
	Liked going to school overall	0.623***
	Felt schoolwork is meaningful	0.713***
	Felt things learned in past year will be important	0.675***
	Felt courses at school are interesting	0.697***
	Teacher says I am doing a good job	0.536***
Depression (Cronbach’s α = 0.97)		
	Sad/empty/depressed most of day or discouraged	0.937***
	Lost interest/pleasure in most things	0.916***
	Changes in appetite or weight	0.848***
	Sleep problems	0.955***
	Others noticed they are restless or lethargic	0.646***
	Felt tired/low energy nearly every day	0.947***
	Felt worthless nearly every day	0.813***
	Inability to concentrate or make decisions	0.952***
	Any thoughts or plans of suicide	0.845***

*** *p* < 0.001

### Structural model

**Fig. 1:**
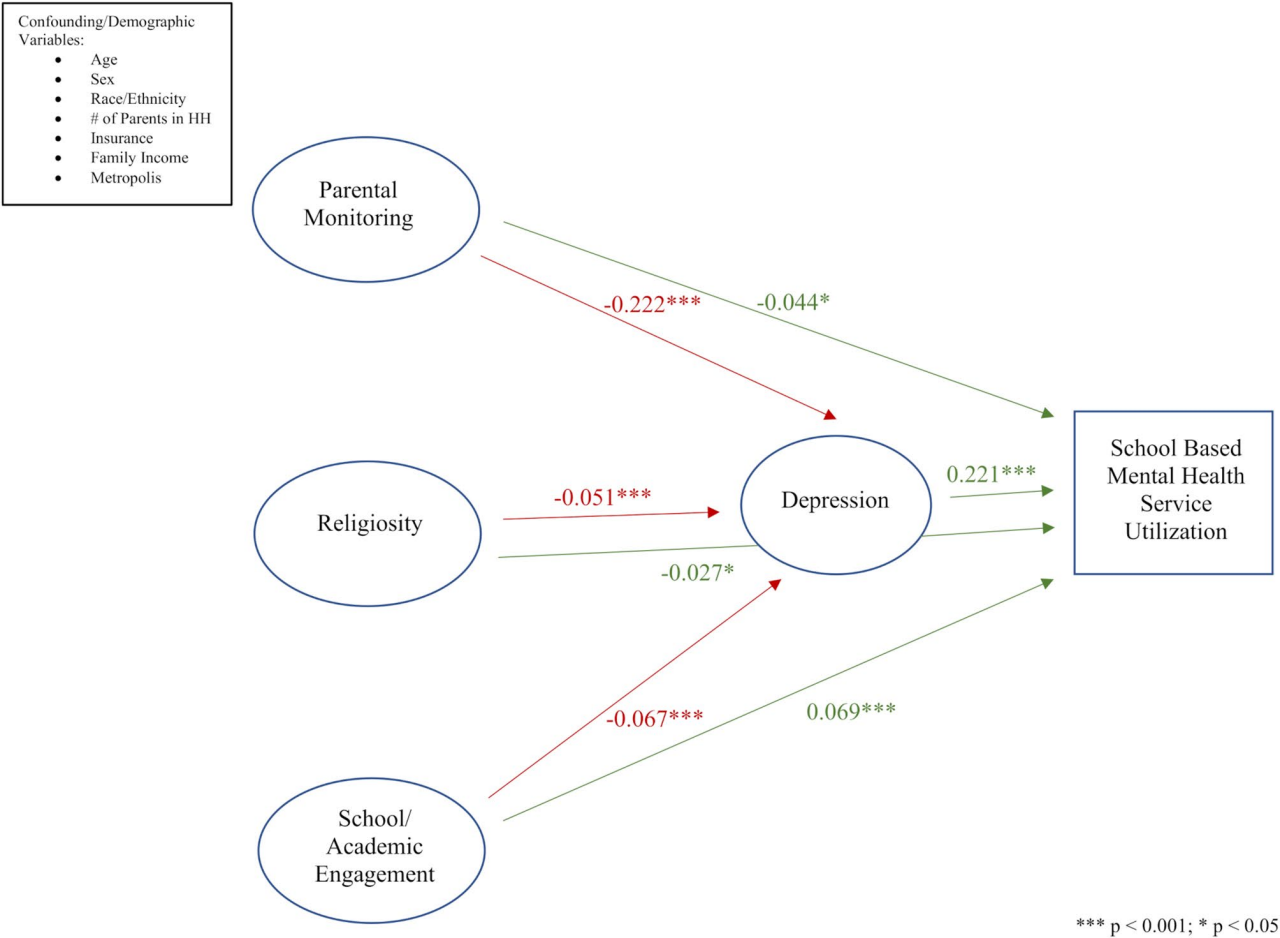
Structural Equation Model

Figure 1 shows all paths of the SEM. Goodness of fit indices for the SEM specify a well fit model and included a CFI equal to 0.930, a TLI equal to 0.923, a RMSEA equal to 0.029, and a SRMR equal to 0.045. Therefore, model respecification was not necessary. The SEM shows that parental monitoring and support (ß = -0.044, *p* < 0.05), religiosity (ß = -0.027, *p* < 0.05), and school and academic engagement (ß = 0.069, *p* < 0.001) have a statistically significant direct effect on SBMHS use. This indicates that adolescents who reported higher levels of parental monitoring and support and religiosity used SBMHS less than adolescents who reported lower levels of parental monitoring and support and religiosity. Relatedly, adolescents who reported higher levels of school and academic engagement used SBMHS more than their counterparts who reported lower levels. Figure 1 also depicts that the effects of parental monitoring and support, religiosity, and school and academic engagement on SBMHS use are mediated by depression. Even though adolescents reporting higher levels of parental monitoring and support (ß = -0.222, *p* < 0.001), religiosity (ß = -0.051, *p* < 0.001), and school and academic engagement (ß = -0.067, *p* < 0.001) are significantly associated with lower levels of depression, the indirect effect of depression (ß = 0.221, *p* < 0.001) shows that adolescents reporting more depressive symptoms use SBMHS more.

## Discussion

The objective of this study was to analyze the relationships between school and academic engagement, religiosity, self-reported depressive symptoms, and parental monitoring and support with adolescent utilization of SBMHS. The results demonstrated statistically significant direct effects of school and academic engagement, religiosity, and parental monitoring and support on SBMHS use among adolescents in addition to mediation of this influential path through self-reported depressive symptoms. The findings support all three of the following proposed hypotheses: (1) higher levels of parental monitoring and support and religiosity along with lower levels of school and academic engagement are likely to decrease adolescent utilization of SBMHS; (2) adolescents who reported a higher number of depressive symptoms are more likely to utilize SBMHS compared to their counterparts who report a lower number and; (3) depression mediates the effects of parental monitoring and support, religiosity, and school and academic engagement on SBMHS utilization.

To the best of our knowledge, this is the first study to analyze the relationships between adolescent utilization of SBMHS and all latent variables individually *and* collectively. As noted in a scoping review, prior studies, however, have examined the effects of each variable separately [24]. Consistent with previous research, the first hypothesis was verified in the direct effect model which posited higher levels of parental monitoring and support and religiosity along with lower levels of school and academic engagement were likely to decrease adolescent utilization of SBMHS. One possible explanation for this significant relationship is that adolescents reporting higher levels of parental support may be more likely to have access to primary or additional mental health services outside of the school setting therefore influencing SBMHS utilization. Adolescents with more parental monitoring and support have reported higher levels of self-esteem, -control, and -efficacy [52–54] contributing to positive mental health overall, potentially decreasing the need and therefore use of SBMHS. Furthermore, adolescents who reported higher levels of parental monitoring and support experienced fewer short- and long-term depressive symptoms [55, 56] which supports this study’s third hypothesis that depressive symptoms mediate the relationship between latent indicators (parental monitoring and support, religiosity, and school and academic engagement) and adolescent utilization of SBMHS. Findings from this study also align with previous literature showing that mental health service delivery in schools was impeded by lack of parental support [14, 57]. Possible explanations for this are federal and state requirements of parental involvement for adolescent use of SBMHS (in most cases), limited family resources, a lack of parental mental health literacy, or parental denial of their adolescent’s mental health concerns.

Although religiosity has been reported as a barrier to seeking mental health services, results from this study align with literature citing this concept as a protective factor for adolescent mental health and well-being, including 90% of articles from one systematic review [32, 58, 59]. Findings from this study’s mediating effects model support the third hypothesis that depression mediates the effects of parental monitoring and support, religiosity, and school and academic engagement on SBMHS utilization. While no research could be found reinforcing the first hypothesis, the direct effects model provides foundational information for further studies. It is possible religion’s structure might serve as guidance for an adolescent when making impactful decisions, testing personal boundaries, or attempting to understand more abstract concepts (e.g., death, hope, or justice) affecting the use of SBMHS.

According to this study’s direct effects model, higher levels of adolescent school and academic engagement are associated with an increase in SBMHS utilization, confirming the third hypothesis of this study. Previous research has also shown that negative school experiences are often associated with increased reports of adolescent depression, thereby supporting this study’s mediating effects model [60, 61]. An adolescent’s perception of a positive school climate, level of involvement in school activities, and development of trusting relationships with teachers, other school personnel, and peers in the educational setting are all associated with an increase in adolescent socio-emotional health and when required, SBMHS utilization [54, 62, 63]. Offering mental health services inside the school building increases accessibility and decreases the need for transportation. Additionally, the close physical proximity increases awareness among students and school personnel that SBMHS are an available resource. Further research is needed to explore which school related factors (e.g., student-teacher ratios; class offerings; specific employee groups such as teachers, administrators, or guidance counselors; after school programming; peer relationships in the classroom) might have the biggest impact on increasing and optimizing SBMHS use.

Although the body of evidence about depression and adolescent use of SBMHS varies, this study’s analysis verifies the second hypothesis, that adolescents who reported a higher number of depressive symptoms are more likely to utilize SBMHS compared to their counterparts who report a lower number, and coincides with findings from Green et al. who found that adolescents reporting depression were 1.7 times more likely to use school counseling services than adolescents who denied depressive symptoms (2020). While depression is the only mental health condition analyzed in this study, it is one of the leading mental health concerns among adolescents [64, 65] and a major reason for seeking out SBMHS [16, 29]. Future studies are needed to explore other factors (e.g., clinical diagnostician; which depressive symptoms were reported; treatments attempted outside of school) that may be influencing current conflicting evidence on the relationship between adolescent depression and SBMHS use.

Overall, this study adds to previous knowledge about specific determining factors associated with adolescent utilization of SBMHS offering insights into roles played by school and academic engagement, religiosity, and parental monitoring and support. Results from this study reinforce the importance of interdisciplinary collaboration by pointing to the complexities involved in an adolescent’s use of SBMHS. This study highlights the importance of teachers, parents, school personnel, and community health practitioners considering the confluence of factors associated with utilization of SBMHS when interacting with or assessing an adolescent’s emotional well-being.

Study results should be considered in the context of its methodological limitations. Although this study analyzed a large, nationally representative dataset enhancing generalizability, neither temporal nor causal relationships cannot be drawn as the NSDUH is cross-sectional or collected at one point in time. Specific to this study and inherent to secondary data analyses, is that indicators for additional factors associated with adolescent utilization of SBMHS may not have been collected and may therefore be missing from the model. Examples beyond this scope of this study may include mental health conditions other than depression, indicators of stigma, socio-political influences, adverse childhood experiences, or larger systems of discrimination and implicit bias. Further studies are required to explore how factors missing from this dataset could affect these results. Recall, social desirability, and response biases may also limit findings due to the nature of self-reported data. Next, this study examined adolescents who reported receiving school specific mental health services, but it was not determined if they were engaged in mental health services outside of the school setting therefore limiting this study’s conclusions. Another limitation is that this study used pre-pandemic data from 2019. It is possible the same analysis using post-pandemic data could reveal different quantitative results in terms of magnitude, direction, or both. The current study serves as a baseline for comparison of future research on factors associated with adolescent utilization of SBMHS. Race and ethnicity groups were combined for model stability which could affect results. This choice eliminated the ability to analyze certain nuances, especially for NSDUH participants placed into the “other” race/ethnicity category. Lastly, structural equation modeling techniques have greater statistical power than traditional multiple regression analyses, but results must be interpreted with the understanding that parental monitoring and support, religiosity, and school and academic engagement, this study’s latent variables, are not pure representations of each construct like measured variables [66].

Despite the aforementioned limitations, several implications can be drawn from this study’s results. Teachers spend most of the school day with students and often observe problematic behavior first [62, 67]. Therefore, developing and delivering high quality training programs to educate teachers, school administrators, and other key personnel about the purpose of SBMHS, specific services offered, and the referral process might enhance their sense of preparedness when recommending a student for mental health assessment or treatment. Relatedly, this study reinforces the importance of encouraging periodic interdisciplinary collaboration meetings between educators, healthcare practitioners, and families [15, 68, 69]. Often, a disconnect between those involved may lead to lapses in treatment, communication breakdowns, and a disengaged adolescent. There is also a shortage of SBMHS providers and those who practice have reported an inability to treat students in need due to the principal focus on assessments for reimbursement and payment purposes [70–72]. This study substantiates the need for policymakers to allocate funds towards increasing the SBMHS workforce by offering economic initiatives in the form of program or certificate tuition reimbursement. Lastly, results support increasing public and private funding opportunities to conduct and disseminate research on feasibility, effectiveness, and implementation of innovative, evidence based SBMHS optimizing adolescent unitization. Not only is research important for education and collaborative practice, but also serves as a core component of children’s mental health policymaking [73, 74].

## Conclusion

Due to the growing number of adolescents who are diagnosed, report experiencing, or are at risk for a mental health disorder, a comprehensive understanding of factors associated with the use of an effective countermeasure, SBMHS, is critical. This study developed an integrated statistical model demonstrating the direct effects of parental monitoring and support, religiosity, and school and academic engagement and mediating effects of depressive symptoms on SBMHS utilization. This SEM quantitatively explains each factor associated with SBMHS use among adolescents alerting psychologists, teachers, counselors, school nurses, and administrators to specific determinants of service requirements. Findings from this study bring to light the influential factors that should be considered when developing strategies for increasing adolescent use of SBMHS, encouraging overall well-being, and actively preventing mental health problems in adulthood.

## Supplementary Information

Below is the link to the electronic supplementary material.

Supplementary Material 1

## Data Availability

No datasets were generated or analysed during the current study.
