# Supplementary material for: Adolescent utilization of school based mental health services in the United States
Source: Int J Ment Health Syst. 2025 Aug 20;19:27. doi: 10.1186/s13033-025-00684-8 (PMC12366107; doi:10.1186/s13033-025-00684-8)
Supplement: Supplementary file 1 — Supplementary Material 1 [file 13033_2025_684_MOESM1_ESM.docx]

Correlations between scales of religiosity, parental monitoring and support, school and academic engagement, depression, and school mental health service utilization were assessed using Pearson’s correlation. Correlations for all latent variable scales and SBMHS are presented in the Supplemental Table 1. Religiosity was positively correlated with parental monitoring and support and school and academic engagement. Religiosity was also negatively correlated with depression and SBMHS use. Depression was negatively correlated with all three other scales (religiosity, parental and monitoring and support, and school and academic engagement) indicating that adolescents with more depressive symptoms reported lower levels of religiosity, parental monitoring and support, or school and academic engagement. While SBMHS use was negatively correlated with religiosity and positively correlated with depression, parental monitoring and support and school and academic engagement were not statistically significant.

________________________________________________________________________________________

Supplemental Table 1. Pearson correlations of latent variable scales and school mental health service use.

|  | Religiosity | Parental Monitoring and Support | School and Academic Engagement | Depression | School Mental Health Service Use |
| --- | --- | --- | --- | --- | --- |
| Religiosity | - |  |  |  |  |
| Parental Monitoring and Support | 0.241*** | - |  |  |  |
| School and Academic Engagement | 0.190*** | 0.383*** | - |  |  |
| Depression | -0.102*** | -0.137*** | -0.175*** | - |  |
| School Mental Health Service Use | -0.031** | -0.011 | 0.002 | 0.229*** | - |

*** p < 0.001; ** p < 0.01
